# Radiogenomics of Stereotactic Radiotherapy: Genetic Mechanisms Underlying Radiosensitivity, Resistance, and Immune Response

**DOI:** 10.3390/genes16070732

**Published:** 2025-06-24

**Authors:** Damir Vučinić, Ana-Marija Bukovica Petrc, Ivona Antončić, Maja Kolak Radojčić, Matea Lekić, Felipe Couñago

**Affiliations:** 1 Department of Oncology and Radiotherapy, Faculty of Medicine, University of Rijeka, 51000 Rijeka, Croatia; anamarija.bukovica@yahoo.com; 2 Special Hospital Radiochirurgia Zagreb, 10431 Sveta Nedelja, Croatia; matea.lekic@live.com; 3 Tumor Clinic, Clinical Hospital Centre Rijeka, 51000 Rijeka, Croatia; ivona.jerkovic051@gmail.com (I.A.); kolak.maja@gmail.com (M.K.R.); 4 Department of Medicine, Faculty of Medicine, Health and Sports, European University of Madrid, 28108 Madrid, Spain; felipe.counago@genesiscare.es; 5 Department of Radiation Oncology, Hospital San Francisco de Asís y La Milagrosa, GenesisCare, 28002 Madrid, Spain

**Keywords:** DNA damage response, immune response, inflammation, precision medicine, radiogenomics, radioresistance, radiosensitivity, stereotactic body radiotherapy

## Abstract

Stereotactic body radiotherapy (SBRT) delivers ablative radiation doses with sub-millimeter precision. Radiogenomic studies, meanwhile, provide insights into how tumor-intrinsic genetic factors influence responses to such high-dose treatments. This review explores the radiobiological mechanisms underpinning SBRT efficacy, emphasizing the roles of DNA damage response (DDR) pathways, tumor suppressor gene alterations, and inflammatory signaling in shaping tumor radiosensitivity or resistance. SBRT induces complex DNA double-strand breaks (DSBs) that robustly activate DDR signaling cascades, particularly via the ATM and ATR kinases. Tumors with proficient DNA repair capabilities often resist SBRT, whereas deficiencies in key repair genes can render them more susceptible to radiation-induced cytotoxicity. Mutations in tumor suppressor genes may impair p53-dependent apoptosis and disrupt cell cycle checkpoints, allowing malignant cells to evade radiation-induced cell death. Furthermore, SBRT provokes the release of pro-inflammatory cytokines and activates innate immune pathways, potentially leading to immunogenic cell death and reshaping the tumor microenvironment. Radiogenomic profiling has identified genomic alterations and molecular signatures associated with differential responses to SBRT and immune activation. These insights open avenues for precision radiotherapy approaches, including the use of genomic biomarkers for patient selection, the integration of SBRT with DDR inhibitors or immunotherapies, and the customization of treatment plans based on individual tumor genotypes and immune landscapes. Ultimately, these strategies aim to enhance SBRT efficacy and improve clinical outcomes through biologically tailored treatment. This review provides a comprehensive summary of current knowledge on the genetic determinants of response to stereotactic radiotherapy and discusses their implications for personalized cancer treatment.

## 1. Introduction

Since its discovery in the late 19th century, ionizing radiation has become central to multidisciplinary cancer care. Modern radiotherapy has evolved to balance effective tumor control with minimal damage to surrounding tissue by optimizing total dose, dose per fraction, number of fractions, and treatment duration. These parameters are carefully calibrated to maximize tumor control while minimizing toxicity to surrounding normal structures. Radiation dose prescription is individualized based on tumor histology, anatomical location, and the tolerance of adjacent healthy tissues [1]. Stereotactic ablative radiotherapy (SABR), also known as stereotactic body radiotherapy (SBRT) for extracranial targets, is a highly precise and advanced radiotherapy modality characterized by the delivery of high doses per fraction (typically >5 Gy) in a limited number of sessions (usually 1–5). When used for intracranial lesions, this approach is termed stereotactic radiosurgery (SRS). Collectively, these techniques fall under the umbrella of stereotactic radiotherapy (SRT), which distinguishes itself from conventional fractionated radiotherapy not only in delivery technique but also in underlying radiobiological mechanisms [2] (Table 1). Traditional radiotherapy has long been guided by the classical “5 Rs” of radiobiology—repair, reoxygenation, redistribution, repopulation, and radiosensitivity. However, mounting evidence suggests that the biological responses triggered by high-dose hypofractionated treatments such as SRT/SBRT transcend these principles, eliciting distinct and often more complex molecular and immunological effects [3]. Notably, SRT has been shown to induce not only direct DNA damage and tumor cell death but also significant remodeling of the tumor microenvironment [3,4,5]. Among the most intriguing findings is its capacity to stimulate systemic anti-tumor immune responses. Recent advances in genomics and molecular biology have ushered in a new era of precision radiation oncology. Genetic profiling is now recognized as an essential component in tailoring radiotherapy protocols to the molecular characteristics of individual tumors [6,7,8]. Both radiosensitivity and resistance are profoundly influenced by germline and somatic genetic alterations affecting pathways involved in DNA damage recognition, repair, and stress responses. Hereditary cancer syndromes involving mutations in key DNA repair genes such as ATM, XRCC2, and RAD51C underscore the central role of these mechanisms in determining tumor and normal tissue responses to ionizing radiation. Moreover, genome-wide association studies (GWAS) continue to uncover novel genes and single-nucleotide polymorphisms (SNPs) associated with variations in treatment efficacy and toxicity [1,8,9]. A comprehensive understanding of the genetic and molecular underpinnings of radiosensitivity, resistance, and immune modulation is critical to further optimizing stereotactic radiotherapy. By integrating high-resolution imaging, precise radiation delivery, immunomodulatory strategies, and individualized genomic insights, radiation oncology is evolving toward a truly personalized approach—one that aims to deliver highly effective and minimally toxic cancer treatments tailored to each patient’s unique biological profile. This review provides a comprehensive summary of current knowledge on the genetic determinants of response to stereotactic radiotherapy. It discusses their implications for personalized cancer treatment, including the potential to improve therapeutic outcomes, reduce treatment-related toxicity, and guide oncologists in tailoring interventions to individual patients.

## 2. Radiobiological Mechanisms of Stereotactic Body Radiation Therapy

### 2.1. Radiobiological Rationale of SBRT and the LQ Model

Stereotactic body radiation therapy delivers high doses in one to a few fractions to small, well-defined target volumes. This hypofractionated approach represents a departure from conventional fractionated radiotherapy, which typically involves lower doses (1–5 Gy per fraction) administered over several weeks, for a total dose ranging from 60 to 70 Gy. Delivering ablative doses per fraction requires exceptional precision in planning, imaging, and dose delivery throughout the radiotherapy process [10]. The radiobiological response to conventional radiotherapy has traditionally been explained by the “4 Rs”: repair of sublethal damage, redistribution of cells in the cell cycle, reoxygenation of hypoxic tumor regions, and repopulation of surviving tumor cells [11]. A fifth R—intrinsic radiosensitivity—was later added to account for individual variations in tumor response [12]. The most widely used model to describe tissue response to radiation is the linear-quadratic (LQ) model, which estimates the surviving fraction of cells as an exponential function of dose, incorporating both a linear component (α) for direct DNA damage and a quadratic component (β) for damage resulting from the interaction of sublethal events. The α/β ratio derived from this model helps characterize the radiosensitivity of different tissues and is instrumental in calculating the biologically effective dose (BED), enabling comparison between fractionation schedules [2]. While the LQ model is well validated for conventional dose ranges (1–6 Gy per fraction), its applicability at higher doses—such as those used in SBRT—remains controversial, especially above 8–10 Gy per fraction [12,13].

### 2.2. Limitations of the LQ Model and Treatment Delivery Impact

**Figure 1:**
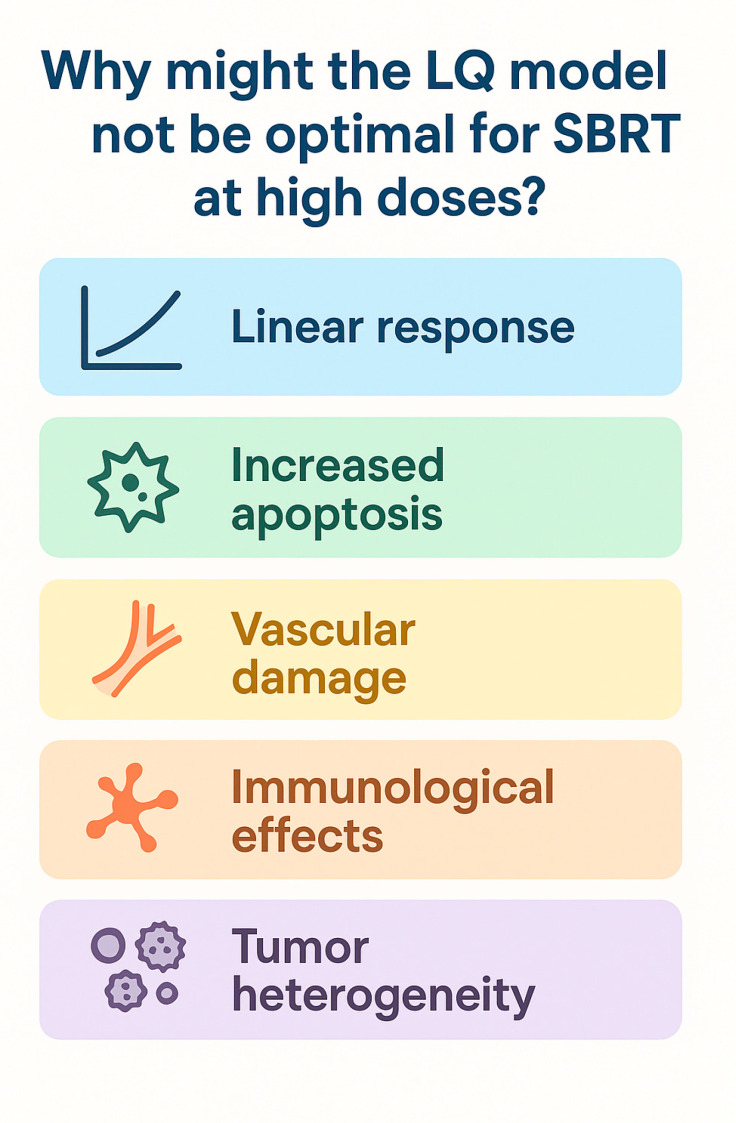
Illustrates the limitations of the classical LQ model at ablative dose levels. At such high doses, the radiation response may become effectively linear, and additional biological effects—including endothelial damage, immune activation, and tumor heterogeneity—further diverge from LQ-based predictions.

Preclinical studies suggest the model may underestimate the biological effects of large single doses, as it does not fully capture complex mechanisms such as vascular damage, immune activation, or cell death beyond mitotic catastrophe [14,15,16]. High-dose radiotherapy may also be affected by treatment delivery time. When treatment duration exceeds 30 min, a reduction in biological effectiveness of approximately 10% can occur, possibly due to sublethal damage repair during prolonged exposure [3,4,17]. Nonetheless, several studies support the validity of the LQ model up to 15–20 Gy per fraction, including experimental evidence that shows single high-dose irradiation induces consistent tumor cell and vascular responses in both in vitro and in vivo settings, thereby supporting the model’s applicability within this dose range [2,17]. Moreover, irradiation with a single high dose (e.g., 20 Gy) can arrest cells in specific cell cycle phases, particularly G1/S, or push cells into the G2 phase, where radiosensitivity increases, before eventual cell death [3]. Unlike conventional schedules, where tumor repopulation typically begins 3–4 weeks into treatment, SBRT’s short duration limits the window for significant repopulation. However, early repopulation cannot be entirely excluded [3]. Another important consideration is reoxygenation: while conventional fractionation allows hypoxic cells to reoxygenate between sessions, SBRT’s limited number of fractions restricts this advantage. Although delivering 6–8 fractions may capture some reoxygenation benefit, it remains less effective than conventional regimens. Extending the interval between SBRT sessions has been proposed as a strategy to promote reoxygenation and enhance treatment efficacy [17]. Indeed, the LQ model tends to underestimate the true therapeutic impact of SBRT, as it primarily accounts for direct DNA damage while overlooking indirect mechanisms such as immune modulation and vascular disruption [4] (Figure 1). High single-dose radiation can overwhelm DNA repair mechanisms, leading to extensive DNA damage. At the same time, these doses may profoundly alter the tumor microenvironment by damaging tumor vasculature and activating innate and adaptive immune responses [12]. The immunomodulatory effects of SBRT are strongly dose- and schedule-dependent. High-dose fractions tend to stimulate cytotoxic immune responses, whereas lower doses (<10 Gy) may exert immunosuppressive effects [18]. Mathematical modeling is increasingly being integrated into radiotherapy research to optimize treatment outcomes by incorporating data from functional imaging, radiomics, deep learning, genomics, and immunological profiling, including single-cell sequencing and liquid biopsies. For instance, the TRIPOD framework has been used to combine imaging-based tumor hypoxia data with radiation dose distribution to predict treatment response and personalize radiotherapy in non-small cell lung cancer patients, demonstrating the clinical potential of integrating mechanistic modeling with patient-specific biological inputs [12]. These advances support a more personalized, biologically guided approach to radiotherapy, enabling the design of treatments tailored to the unique characteristics of each patient’s tumor.

## 3. Genetic Factors and Stereotactic Body Radiation Therapy Resistance

### 3.1. DNA Damage Repair Upregulation

One of the most critical determinants of tumor radioresistance is the ability to recognize and repair DNA damage. High-dose hypofractionated radiation, such as SBRT, induces numerous DNA double-strand breaks (DSBs) that can overwhelm a cell with normal repair capacity. However, radioresistant tumors often upregulate key components of the DNA damage response (DDR) and repair pathways, allowing for more efficient resolution of lethal DNA lesions [19]. In many radioresistant cancer cells, the non-homologous end-joining (NHEJ) pathway, the primary mechanism for repairing double-strand breaks (DSBs), is particularly active. Overexpression or hyperactivation of the DNA-dependent protein kinase catalytic subunit (DNA-PKcs, encoded by the *PRKDC* gene) and other non-homologous end-joining (NHEJ) factors have been observed in radioresistant phenotypes [11,20]. Similarly, the ATM (ataxia telangiectasia mutated) kinase, a master regulator of the DSB response, is frequently overexpressed or constitutively active in resistant tumors. Preclinical studies have shown that cancer cells made radioresistant through repeated irradiation evolve higher ATM levels and faster DNA repair kinetics [21]. For example, studies in murine models have demonstrated that ATM-deficient neural tissues exhibit enhanced sensitivity to ionizing radiation, supporting a radiosensitizing effect that may be leveraged while simultaneously protecting normal brain structures through differential engagement of the DDR pathway [20]. In contrast, ATM inhibition resensitizes these cells to ablative doses of radiation. Clinically, ATM mutations in tumors can have a nuanced impact on response, in some cases creating a DNA repair defect that increases radiosensitivity, while often inducing compensatory pathways that maintain resistance (for instance, ATM-deficient cells relying on alternative repair via PARP). Several trials are investigating ATM or ATR kinase inhibitors in combination with SBRT to leverage DDR dependencies in tumors. Initial findings suggest that inhibiting ATM can significantly enhance the effectiveness of SBRT, particularly with larger fractional doses. For example, the deletion of ATM or its pharmacologic inhibition has allowed a single 15 Gy dose to ablate otherwise radioresistant tumors in preclinical models [22].

**Figure 2:**
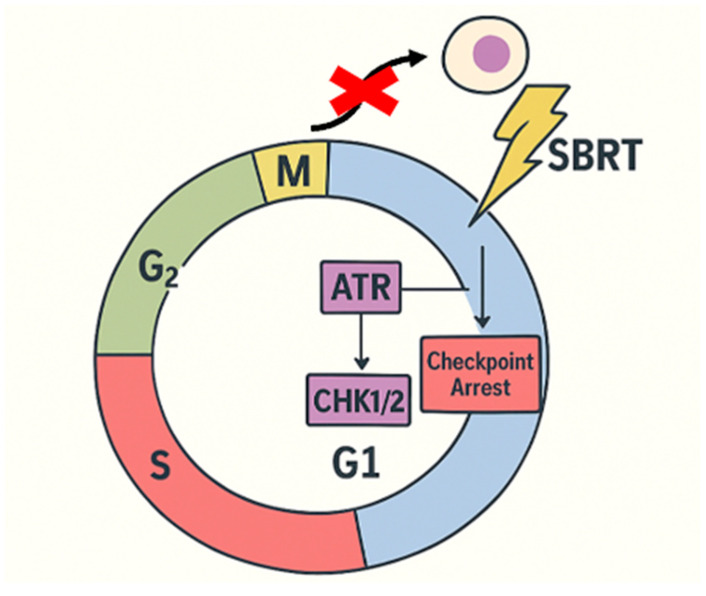
Schematic illustration of how constitutive ATR activation and CHK1/2 overexpression promote G_2_/M checkpoint arrest in response to high-dose SBRT, enabling tumor cells to avoid mitotic catastrophe.

Another adaptation of tumors to high-dose radiation is the robust activation of cell-cycle checkpoints (ATM/ATR–CHK1/2 pathways), which prolongs the G2/M arrest and allows cells time to repair their DNA before mitosis. Tumors with constitutive ATR activation or overexpression of checkpoint proteins can thus evade mitotic catastrophe after SBRT (Figure 2). This provides a rationale for combining checkpoint kinase inhibitors with hypofractionated radiation [23]. In summary, tumors that survive SBRT often do so by amplifying the mechanisms that usually maintain genomic integrity—a double-edged sword that can be targeted. Therapeutically, exploiting this dependency using DDR-targeted agents (ATM, ATR, DNA-PKcs, or PARP inhibitors) is a promising strategy to overcome resistance, as it can tip the balance between the extensive DNA damage induced by SBRT and the tumor’s capacity to repair it. Precision radiation oncology efforts are increasingly focused on identifying these DDR upregulations in patients and personalizing treatment accordingly—for example, delivering higher BED if a tumor’s radiogenomic profile predicts hyper-efficient DNA repair, or adding a radiosensitizing drug to nullify the repair advantage.

### 3.2. Tumor Suppressor Mutations and Clonal Selection

Loss-of-function mutations in key tumor suppressor genes represent a fundamental mechanism of resistance to SBRT. These mutations impair essential cellular responses to DNA damage, including cell cycle arrest, apoptosis, and senescence, enabling malignant cells to survive doses of radiation that would otherwise be lethal. The most prominent example is *TP53*, which encodes the tumor suppressor protein p53, often referred to as the “guardian of the genome” [23]. In response to radiation-induced DNA damage, wild-type p53 activates transcriptional programs that induce cell cycle arrest or trigger apoptosis when damage is irreparable. Disruption of p53 function abolishes these protective responses, allowing continued cell proliferation despite genomic instability. Clinically, *TP53* mutations are strongly associated with radioresistance and local treatment failure across multiple cancer types. In a landmark study in head and neck squamous cell carcinoma, tumors harboring disruptive *TP53* mutations demonstrated significantly higher rates of local recurrence following radiotherapy, likely due to defective radiation-induced senescence and apoptotic signaling [24]. Preclinical studies have shown that restoring p53 function in TP53-mutant tumors can resensitize cells to radiation by reactivating apoptotic and antiproliferative pathways [4,25]. However, in tumors with complete loss or non-functional mutations of *TP53*, alternative approaches—such as targeting compensatory survival pathways or delivering higher biologically effective doses—are necessary to overcome resistance. Other tumor suppressors commonly inactivated in radioresistant tumors include SMAD4 and PTEN. Loss of SMAD4 has been shown to increase intracellular reactive oxygen species (ROS) levels and autophagic flux following radiation, enhancing tumor cell resilience to oxidative stress. Meanwhile, PTEN loss, common in glioblastomas and melanomas, removes a critical negative regulator of the PI3K/AKT pathway, resulting in constitutive pro-survival signaling. Activated AKT promotes radiation resistance by stabilizing anti-apoptotic proteins and accelerating DNA repair processes. In preclinical models, AKT inhibition or PTEN restoration resensitizes tumors to radiation [26]. More broadly, high-throughput sequencing of post-radiotherapy tumor samples supports a clonal selection model, in which radiation eliminates sensitive tumor clones while enriching for pre-existing subclones harboring mutations in genes like TP53, PTEN, and LKB1. For example, rectal cancer organoid studies have traced radioresistant outgrowths to rare subclones present before treatment [27]. The intense selective pressure exerted by SBRT may similarly drive the expansion of resistant clones, particularly in genetically heterogeneous tumors. These findings highlight the potential utility of radiogenomic profiling prior to treatment to identify high-risk subclonal populations. Such insights could guide adaptive strategies, including dose intensification or incorporating targeted agents to overcome intrinsic resistance [28]. Ultimately, tumor suppressor mutations allow malignant cells to evade DNA damage checkpoints and resist cell death, often by co-opting parallel pro-survival pathways such as AKT or NRF2. These alterations represent actionable vulnerabilities that may be exploited using combination therapies designed to disable compensatory mechanisms and restore sensitivity to SBRT.

### 3.3. Inflammatory Signaling

**Figure 3:**
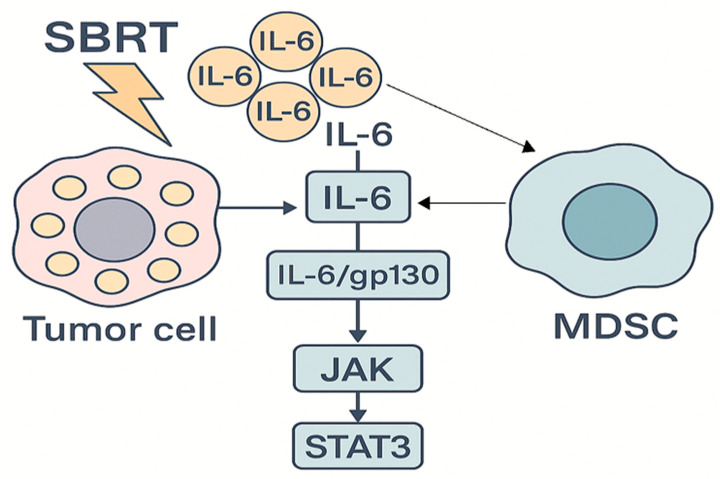
Secreted IL-6 binds the IL-6R/gp130 receptor complex on both tumor cells and infiltrating myeloid-derived suppressor cells (MDSCs), leading to activation of JAK kinases and subsequent phosphorylation of STAT3. Activated STAT3 drives transcriptional programs that promote MDSC recruitment, establishing a feed-forward loop that sustains STAT3 signaling and fosters an immunosuppressive microenvironment, thereby contributing to tumor radioresistance.

Chronic activation of inflammatory signaling pathways in the tumor microenvironment has emerged as a critical contributor to radioresistance in the context of stereotactic radiotherapy. Ionizing radiation triggers DNA damage and a complex cascade of biological responses, including the release of cytokines, recruitment of immune cells, and activation of transcription factors such as NF-κB and STAT3 [23]. While these processes may promote anti-tumor immunity under certain conditions, they can also paradoxically support tumor survival and repair after SBRT. The interleukin-6 (IL-6)/STAT3 signaling pathway is a key axis implicated in this adaptive resistance. IL-6 is frequently upregulated in aggressive or treatment-resistant tumors and has been shown to promote radioresistance by activating STAT3-mediated transcriptional programs. In prostate cancer models, IL-6 exposure enhances ROS scavenging capacity and impairs apoptotic responses, resulting in diminished DNA damage and increased survival following irradiation [1,25,29]. Conversely, inhibition of IL-6—either through neutralizing antibodies or genetic knockdown—restores radiosensitivity by increasing oxidative stress, DNA damage, and apoptotic signaling. In vivo, IL-6 blockade reduces angiogenesis and recruitment of immunosuppressive myeloid-derived suppressor cells (MDSCs), slowing tumor regrowth after SBRT [25,30] (Figure 3). Similarly, NF-κB plays a central role in orchestrating inflammation-induced radioresistance. Radiation activates NF-κB via ATM- or PARP-dependent pathways in both tumor and stromal cells. Once activated, NF-κB induces the expression of a broad array of cytoprotective genes, including anti-apoptotic proteins (e.g., BCL-2, IAPs), antioxidants (e.g., SOD2, catalase), and DNA repair enzymes [9,18]. Inhibiting NF-κB activation sensitizes tumors to radiation and has been shown to disrupt adaptive resistance in experimental models [21]. The influence of NF-κB extends beyond tumor cells: in cancer-associated fibroblasts (CAFs), radiation induces secretion of pro-inflammatory cytokines (e.g., IL-6, IL-1β, TNF-α) in an NF-κB-dependent manner, creating a supportive niche for tumor cell survival. Additionally, TGF-β, classically regarded as an immunosuppressive cytokine, is also upregulated following high-dose radiation and contributes to fibrosis, tissue remodeling, and immune evasion. TGF-β signaling can upregulate DDR genes such as *ATM*, enhancing DNA repair and promoting cell survival. It also recruits immunosuppressive macrophages and regulatory T-cells, further dampening the immune response elicited by SBRT [25,26,27]. Given its multifaceted role in promoting radioresistance, TGF-β is being explored as a therapeutic target in combination with SBRT, particularly in tumors with baseline high TGF-β activity, such as pancreatic cancer [25,28]. Collectively, inflammatory signaling networks support tumor persistence under ablative radiation stress by limiting oxidative damage, inhibiting apoptosis, modulating DNA repair, and fostering an immunosuppressive microenvironment. Targeting these pathways—using IL-6/STAT3 or NF-κB inhibitors, COX-2 blockers, or TGF-β antagonists—represents a promising approach to enhance SBRT efficacy [4,19]. Clinical trials are currently evaluating these strategies, often in combination with immune checkpoint inhibitors, to transform the tumor-promoting inflammatory milieu into a tumoricidal immune response. Moreover, profiling tumors for inflammation-related gene signatures (e.g., IL-6, TNF-α, NF-κB activity) may help identify patients most likely to benefit from anti-inflammatory radiosensitizing therapies.

## 4. Genetic Determinants of SBRT Resistance in Solid Tumors

### 4.1. Non-Small Cell Lung Cancer (NSCLC)

**Figure 4:**
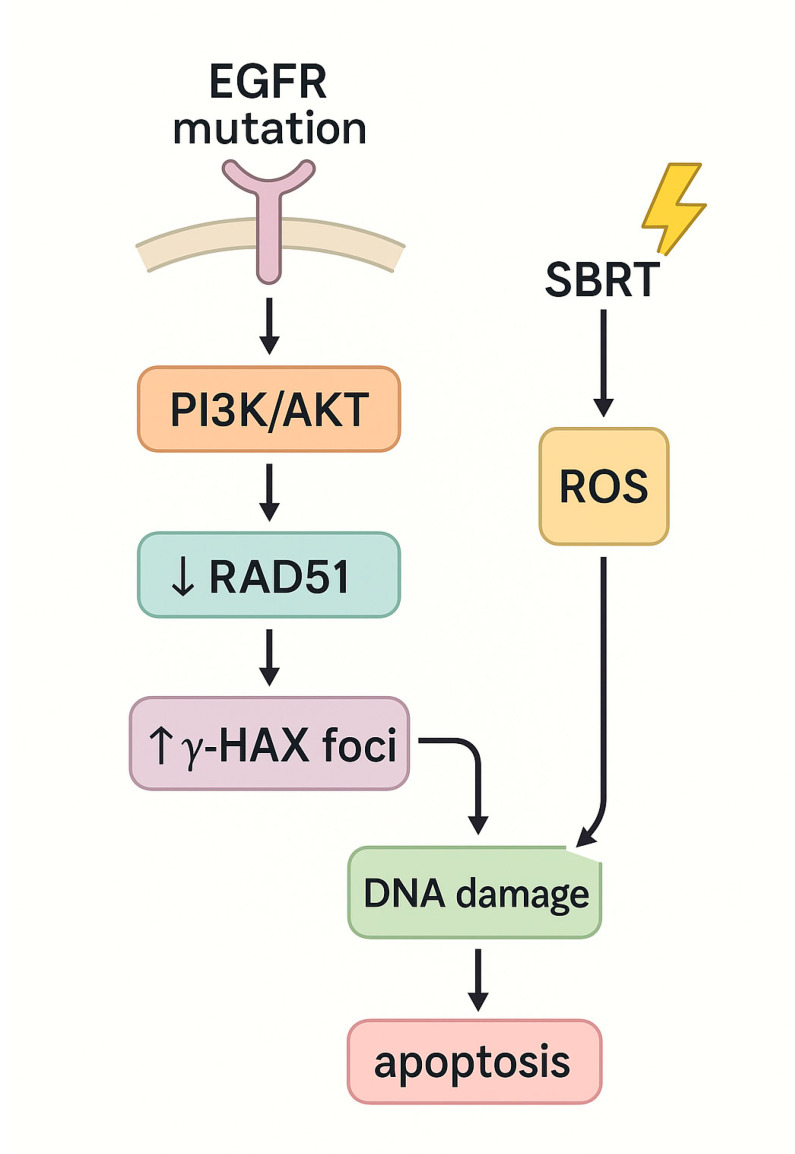
Schematic representation of enhanced SBRT sensitivity in *EGFR*-mutant NSCLC. Activating *EGFR* mutations downregulate the homologous recombination protein RAD51 via PI3K/AKT signaling, increasing γ-H2AX DNA damage foci. Concurrent SBRT-induced ROS further amplify DNA damage, culminating in apoptosis.

Non-small cell lung cancer (NSCLC) remains the most prevalent and lethal malignancy worldwide, posing a major global health burden. In 2022, lung cancer remained the leading cause of cancer death globally, with an estimated 1.8 million deaths. It was also the most frequently diagnosed cancer, accounting for approximately 2.5 million new cases, or 12.4% of all cancers [31,32]. In recent years, SBRT has revolutionized the management of early-stage and oligometastatic NSCLC by offering high rates of local control with a non-invasive approach [33,34,35]. Despite these advantages, a subset of tumors exhibit resistance to SBRT, often driven by distinct genetic alterations. However, the emergence of resistance to SBRT in a subset of tumors underscores the need to elucidate the genetic mechanisms driving this phenomenon [36]. Genomic studies have identified several recurrent mutations associated with radioresistance in NSCLC, most notably in the KEAP1-NFE2L2 (NRF2) axis. Under normal conditions, KEAP1 binds to NFE2L2 and targets it for degradation via CUL3-mediated ubiquitination. In response to oxidative or electrophilic stress, KEAP1 releases NFE2L2, which translocates to the nucleus and induces the transcription of antioxidant response genes. Inactivating mutations in *KEAP1* or activating mutations in *NFE2L2* lead to constitutive activation of this pathway, allowing tumor cells to neutralize reactive oxygen species (ROS) and withstand oxidative stress induced by SBRT or systemic therapy [37,38,39,40,41,42,43,44,45,46,47]. These mutations are found in approximately 20% of NSCLC tumors and are strongly associated with poor local control after definitive radiotherapy, including SBRT [40,41]. Preclinical models have demonstrated that KEAP1-deficient NSCLC cells exhibit increased glutathione levels and decreased DNA damage following radiation. However, treatment with glutaminase inhibitors (such as CB-839, a glutaminase inhibitor)—agents that deplete glutathione—restores radiosensitivity, suggesting a viable therapeutic strategy for this genetic subset [42,43,44]. Another key genetic alteration is the loss of *STK11*, which encodes liver kinase B1 (LKB1), the second most frequently mutated tumor suppressor in NSCLC (17–23% of cases) [44]. *STK11* mutations often co-occur with activating *KRAS* mutations and are associated with aggressive tumor behavior, immunosuppressed phenotypes, and reduced response to therapy [45]. Recent studies indicate that the KEAP1/NRF2 axis plays a critical role in mediating radioresistance in LKB1-deficient tumors, and that targeting glutamine metabolism may overcome this resistance [43,46]. LKB1 loss leads to metabolic reprogramming, wherein tumor cells shift from glucose metabolism to reliance on glutamine for energy, rendering them sensitive to glutamine deprivation strategies. KRAS mutations, present in a substantial proportion of NSCLC cases, are also linked to resistance to SBRT. Retrospective analyses of patients undergoing definitive radiotherapy, including SBRT, have shown decreased local progression-free survival in KRAS-mutant tumors, even after adjusting for confounding variables [44,46]. Mechanistically, *KRAS*-mutated tumors exhibit upregulation of pro-survival signaling pathways (e.g., MAPK/ERK, PI3K/mTOR) and enhanced DNA repair capacity, contributing to their resilience against radiation [47]. These findings support the rationale for combining SBRT with targeted agents such as DNA repair inhibitors or MEK inhibitors in this molecular subset. *TP53* mutations, present in approximately 50% of NSCLC cases, further contribute to radioresistance by disrupting key regulatory processes such as DNA repair, apoptosis, and cell cycle arrest [3,48]. Multiple studies have demonstrated poorer outcomes in TP53-mutant NSCLC following SBRT, including higher recurrence rates and reduced overall survival [45,46,47,48]. Although the mechanism remains to be fully elucidated, it has been proposed that TP53 loss may attenuate oxidative stress signaling or restore apoptotic thresholds in KEAP1-mutant tumors, thereby paradoxically improving SBRT response. Interestingly, one study by Saleh et al. found that co-occurring *TP53* mutations in *KEAP1*-mutant tumors were paradoxically associated with improved survival, suggesting complex interactions between these pathways. Conversely, *EGFR* mutations—among the most common driver alterations in NSCLC—are associated with increased radiosensitivity. *EGFR*-mutant tumors generally exhibit longer overall survival and superior responses to tyrosine kinase inhibitors (TKIs). In the context of radiotherapy, *EGFR* mutations have been linked to better local control and reduced recurrence rates [49,50]. Mechanistically, *EGFR* activation has been shown to downregulate RAD51, a key homologous recombination factor, resulting in impaired DNA repair and increased radiation-induced DNA damage. Additionally, SBRT-induced ROS generation further exacerbates this damage, promoting apoptosis (Figure 4) [49]. Emerging evidence also suggests that SBRT may delay the onset of acquired resistance to EGFR-TKIs and prolong survival in EGFR-mutant NSCLC, supporting its integration into multimodal treatment strategies [50]. These findings underscore the importance of molecular profiling in guiding SBRT use and highlight potential radiosensitization strategies based on tumor genotype.

### 4.2. Pancreatic Ductal Adenocarcinoma (PDAC)

Pancreatic ductal adenocarcinoma (PDAC) remains one of the most lethal malignancies, with over 62,000 new cases reported in 2022 alone [32]. Despite therapeutic advances, PDAC is notoriously difficult to treat due to late-stage diagnosis, limited eligibility for surgical resection, and poor long-term survival outcomes. Only approximately 20% of patients are candidates for curative-intent surgery, while an additional 30% present with locally advanced PDAC (LAPC), often precluding resection due to vascular invasion [51]. SBRT has emerged as a promising strategy in LAPC, offering precise delivery of high-dose radiation over a short treatment course. It has demonstrated potential benefits in local control and treatment convenience, particularly when integrated with systemic chemotherapy. Recent data suggest that higher biologically effective doses (BED) delivered via SBRT may yield superior outcomes compared to conventional radiotherapy in selected LAPC patients [52]. Nonetheless, the therapeutic efficacy of SBRT in PDAC is significantly constrained by both intrinsic and acquired radioresistance. Molecular profiling has uncovered several genetic and cellular pathways implicated in this resistance, including impaired apoptosis, enhanced DNA damage repair, inflammation, and hypoxia. Key signaling pathways—such as PI3K/AKT, Notch, FoxO, ATM/ATR, MEK/ERK, TGF-β, and Wnt—as well as genes like *SMAD4*, *MUC1*, and *RAD54*, are central to modulating radiosensitivity in PDAC [53] (Table 2). While the optimal sequencing of treatment remains debated, current guidelines support upfront systemic chemotherapy (e.g., FOLFIRINOX or gemcitabine-based regimens), followed by SBRT in patients without metastatic progression [54]. Among the genetic drivers of radioresistance, *SMAD4* stands out as a pivotal factor. Frequently deleted or mutated in approximately 55% of PDAC cases, *SMAD4* encodes a core effector of TGF-β signaling and is strongly associated with poor prognosis and resistance to radiotherapy [55]. Studies have shown that *SMAD4*-deficient tumors exhibit elevated ROS and autophagy following radiation exposure, which serve as survival mechanisms [56]. More recently, *SMAD4* was found to interfere with DNA repair by binding to PARP1 via its MH1 domain, thereby impairing efficient response to DNA damage. Notably, combining SBRT with PARP inhibitors such as olaparib in SMAD4-deficient models significantly suppressed tumor growth, identifying a promising radiosensitization strategy. Mutations in *TP53*, present in up to 75% of PDAC cases, also contribute to radioresistance. These mutations impair apoptosis and reshape the tumor microenvironment, promoting inflammation, angiogenesis, and immune evasion [24,57,58]. Mutant p53 may upregulate inflammatory mediators such as IL-6 and TNF-α and enhance VEGF-driven angiogenesis. Preclinical models demonstrate that restoration of wild-type *TP53* function using MDM2 inhibitors (e.g., nutlin-3a) can reinstate T-cell activity and reduce tumor-promoting inflammation, offering another potential radiosensitization strategy [59]. *KRAS* mutations, which occur in over 90% of PDAC tumors, further underpin resistance to radiation. KRAS-mutant cells demonstrate enhanced DNA repair via upregulation of NRF2 and 53BP1, leading to accelerated non-homologous end-joining (NHEJ) repair and prevention of mitotic catastrophe [21,53]. The KRAS-NRF2-53BP1 axis plays a central role in protecting tumor cells from radiation-induced DNA damage. In preclinical models, co-treatment with the *KRAS*^G12D^ inhibitor MRTX1133 and SBRT significantly improved local tumor control and survival, suggesting clinical potential for combination approaches [60,61,62]. Additionally, *ATM* mutations—found in 2–18% of somatic and 1–34% of germline PDAC cases—confer sensitivity to DNA damage due to impaired DSB repair. These tumors often rely on alternative repair mechanisms, rendering them vulnerable to ATR or PARP inhibition [63]. *BRCA1/2* mutations, present in 5–10% of familial and ~3% of sporadic PDAC cases, are associated with homologous recombination deficiency and increased tumor mutational burden. These features confer greater sensitivity to DNA-damaging agents, including platinum-based chemotherapy and PARP inhibitors [64]. DDB2, another emerging biomarker, enhances DNA repair and post-radiation cell survival. Low DDB2 expression may increase responsiveness to PARP inhibition in combination with radiotherapy, although further validation is needed [65].

### 4.3. Renal Cell Carcinoma (RCC)

Renal cell carcinoma (RCC) has long been regarded as a radioresistant malignancy. RCC is considered an immunogenic tumor, capable of eliciting robust immune responses, and has demonstrated durable clinical benefits in a subset of patients treated with immune checkpoint inhibitors. However, the emergence of SBRT is beginning to challenge this perception. A phase II trial by Tang et al., published in The Lancet Oncology in 2021, demonstrated that SBRT is a safe and effective treatment option for RCC, with a median progression-free survival of 22.7 months, no treatment-related mortality, and prolonged freedom from systemic therapy in most patients [66]. These findings support the growing role of SBRT as a potentially practice-changing modality, offering durable disease control while minimizing toxicity and preserving quality of life. Despite this progress, a subset of RCC tumors exhibits resistance to SBRT, even at high radiation doses. Genetic alterations play a central role in mediating this resistance and may also serve as predictive biomarkers for response to therapy. Key mechanisms implicated in SBRT resistance in RCC include enhanced DNA repair capacity, hypoxia signaling via the VHL/HIF axis, and emerging regulators such as DLX5 and autophagy-related genes. Among the most well-characterized genetic determinants of radioresistance is *ATM,* a critical regulator of the DNA damage response. Elevated ATM expression has been observed in radiation-resistant RCC cell lines, where it enhances double-strand break repair through γ-H2AX phosphorylation and homologous recombination [22,67]. Preclinical studies have shown that ATM inhibition—using agents like CP466722—restores radiosensitivity, underscoring ATM’s potential as a therapeutic target. While low ATM expression correlates with poor prognosis in clear cell RCC, its sustained activation in resistant tumors promotes survival by maintaining genomic integrity [68]. Additionally, germline and somatic ATM mutations may contribute to genomic instability and interact with *VHL* mutations to drive resistance [69]. Other DNA repair genes, including *XRCC1*, *XRCC3*, and *RAD51*, are also upregulated in radioresistant RCC models. Northern blot analyses show higher baseline and radiation-induced expression of these genes in resistant cell lines, suggesting an enhanced homologous recombination capacity [70]. While XRCC1 expression is generally reduced in clear cell RCC and associated with invasion and poor survival, its prognostic value remains unclear [71,72]. Conversely, RAD51 upregulation has been strongly linked to worse outcomes and appears to influence both genomic stability and tumor metabolism, including glycolysis [72,73,74]. Hypoxia-related signaling further contributes to RCC radioresistance, particularly through *VHL* inactivation and stabilization of HIF-2α. This transcription factor drives expression of DNA repair genes (e.g., *LOX*, *CXCR4*) and supports metabolic adaptations such as mTORC1 activation via amino acid transporters (e.g., SLC7A5) [75,76,77]. Targeting HIF-2α using small-molecule inhibitors like PT2399 enhances radiation sensitivity by inducing G2/M arrest and disrupting survival signaling. However, resistance mechanisms may emerge through compensatory HIF-1α activation or mutations that stabilize the HIF-2α dimer [75,78,79]. Taken together, these findings position ATM, RAD51, and HIF-2α as central mediators of radioresistance in RCC. Their expression profiles may serve as biomarkers to identify patients less likely to benefit from SBRT alone and guide the use of radiosensitizing agents, such as ATM inhibitors, PARP inhibitors, or HIF-2α antagonists, in combination strategies. Future efforts should focus on integrating radiogenic profiling into clinical practice to stratify patients better and personalize SBRT-based treatments. Such an approach may allow for the rational selection of patients likely to benefit from SBRT monotherapy versus those requiring adjunctive targeted therapies to overcome genetic resistance mechanisms.

### 4.4. Prostate Cancer

Most localized prostate cancers are inherently radiosensitive; however, a subset exhibits resistance to ablative doses of SBRT, primarily due to upregulation of DNA damage response (DDR) pathways. In particular, overactivity of ATM, ATR, and DNA-PKcs (encoded by *PRKDC*) has been implicated in SBRT resistance. Radiation exposure itself can induce a surge in DDR gene expression, including *PRKDC*, enabling tumor cells to repair radiation-induced double-strand breaks [80,81] rapidly.

Homologous recombination (HR)-proficient tumors, such as those with wild-type *BRCA1/2*, depend on intact non-homologous end-joining (NHEJ) and checkpoint signaling to survive radiation. In these cases, combining SBRT with DNA-PK or ATM inhibitors may enhance tumor radiosensitivity [82,83]. Key mechanisms contributing to resistance include:

• Overexpression of DNA-PKcs, which promotes efficient DSB repair via NHEJ.
• Constitutive activation of ATM/ATR signaling, which facilitates prolonged cell cycle arrest and damage repair
• Androgen receptor (AR) signaling, which stabilizes DNA-PK complexes and strengthens the DDR, creating a positive feedback loop [80,81]

**Figure 5:**
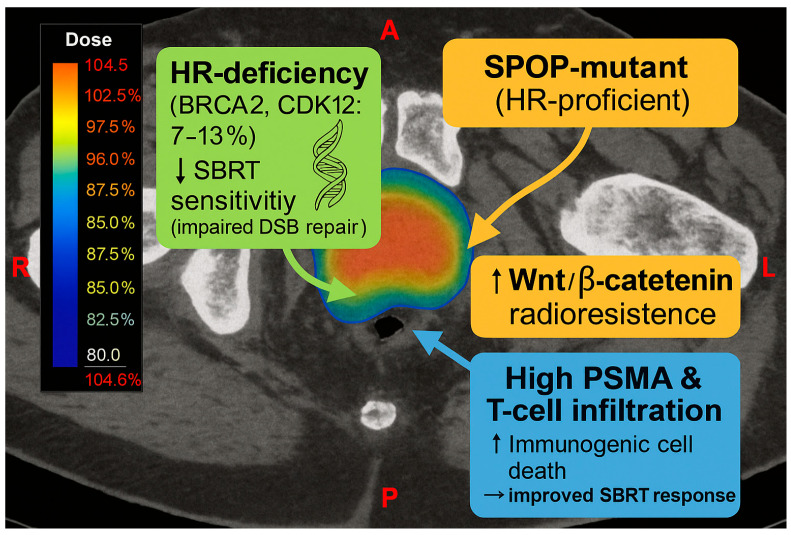
Axial CT slice from a prostate SBRT treatment plan. Tumor subregions demonstrate distinct radiogenomic and immunologic phenotypes. Green: HR-deficient lesions with impaired DSB repair and heightened SBRT sensitivity; Orange: *SPOP*-mutant, HR-proficient regions with Wnt/β-catenin activation and increased radioresistance; Blue: PSMA-high, T-cell–rich areas exhibiting improved SBRT response via immunogenic cell death.

ATM loss-of-function mutations, found in 5–7% of advanced prostate cancers, and constitutive ATR activation further enhance checkpoint responses, enabling evasion of mitotic catastrophe. In parallel, *KEAP1* loss leads to dysregulated NRF2 activity and impaired oxidative stress responses, contributing to radiation resistance [9,82,83]. Mutations in *TP53*, commonly seen in castration-resistant prostate cancer, impair apoptosis and cell cycle arrest, allowing survival despite accumulated DNA damage. When co-occurring with *KRAS* mutations (in approximately 7% of cases), resistance is amplified through the activation of the MAPK and PI3K/AKT pathways [82,84]. Conversely, tumors with homologous recombination deficiencies, such as *BRCA2* mutations (seen in 7–13% of metastatic cases) or CDK12 loss, are more susceptible to SBRT due to defective DSB repair. Nonetheless, not all HR-proficient tumors respond uniformly. For example, *SPOP*-mutant tumors, although maintaining HR functionality, activate Wnt/β-catenin signaling and are associated with increased radioresistance [82,83] (Figure 5). In addition to intrinsic genomic factors, the tumor immune microenvironment influences SBRT outcomes. Prostate tumors with high prostate-specific membrane antigen (PSMA) expression and dense lymphocytic infiltration often show enhanced responses to SBRT, likely due to increased immunogenic cell death [85]. These findings highlight the importance of integrating radiogenomic and immunologic profiling to guide personalized SBRT strategies. Combining SBRT with DDR-targeted agents (e.g., ATM, DNA-PK, or PARP inhibitors) and immunomodulatory approaches holds promise for overcoming resistance and improving outcomes in prostate cancer.

### 4.5. Other Solid Tumors

Patterns of response to SBRT vary significantly across tumor histologies, reflecting distinct underlying genomic and molecular features (Table 3). Tumors historically considered “radioresistant”—such as soft-tissue sarcomas and melanomas—often require higher SBRT doses to achieve comparable local control. For example, a study evaluating SBRT for lung metastases reported 2-year local control rates of ~87% for sarcoma and melanoma metastases, compared to ~100% for breast cancer metastases, underscoring tumor-intrinsic variability in radiosensitivity [86]. Sarcomas typically harbor complex karyotypes and frequently overexpress genes related to DNA repair and cell-cycle progression, which may contribute to intrinsic radioresistance. Melanomas, meanwhile, often present with activating mutations in *BRAF* or *NRAS*, and loss of *PTEN*, promoting pro-survival signaling through MAPK and PI3K/AKT pathways [87]. Similarly, colorectal cancer (CRC) metastases exhibit relatively low local control rates following SBRT. Genomic analyses indicate that metastatic CRC frequently retains functional mismatch repair (MMR) mechanisms and demonstrates EGFR/AKT pathway activation—features associated with chemoresistance and reduced radiosensitivity [88,89,90]. In hepatocellular carcinoma (HCC), SBRT is increasingly used as a local therapy for patients who are ineligible for surgery. While genomic predictors of SBRT response remain under investigation, early studies have explored the potential of liquid biopsy-based biomarkers. In one such study, mRNA profiling of blood samples before and after SBRT in HCC patients identified two candidate genes—*ADIPOR1* and *EPB42*—whose expression changes were associated with treatment efficacy [91]. In particular, ADIPOR1 (adiponectin receptor 1) showed promise: patients with favorable SBRT responses exhibited significantly higher post-treatment ADIPOR1 expression. This marker demonstrated 100% sensitivity and 83% specificity for distinguishing responders, suggesting that circulating gene expression signatures may be noninvasive predictors of SBRT outcomes [90,91]. At the genomic level, HCC is a heterogeneous disease, with common mutations in *TP53*, *CTNNB1* (β-catenin), and *TERT*. Tumors with intact *TP53* and reduced proliferative signaling may be more responsive to SBRT. In contrast, those with Wnt pathway activation or *TP53* mutations may exhibit greater resistance due to aggressive biology and enhanced DNA repair capacity [92]. Collectively, these findings reinforce the critical role of molecular subtype in dictating SBRT response across solid tumors. Tumors harboring aberrations that enhance DNA repair, cell survival, or stress response pathways, such as *KEAP1*, *KRAS*, or *TP53* mutations, are generally more resistant. Conversely, tumors with DDR deficiencies, high tumor mutational burden, or robust immune activation tend to be more SBRT-sensitive.

## 5. Discussion

This review synthesizes current knowledge on the genetic and molecular mechanisms underlying tumor responses to stereotactic body radiation therapy. Three key pathways consistently contribute to SBRT resistance: (1) upregulation of DNA damage repair (DDR) mechanisms, (2) loss-of-function mutations in tumor suppressor genes leading to clonal selection and treatment failure, and (3) pro-tumor inflammatory signaling that modulates both survival and immune evasion. In parallel, tumor-intrinsic factors also influence radiation-induced immunogenicity and radiosensitivity. Collectively, these findings illustrate a complex biological equilibrium where tumor genomics dictate the balance between DNA damage and repair, cell death and survival, and immunogenic versus immunosuppressive radiation effects. A recurring theme is that radioresistant tumors often co-opt cellular stress responses that are normally protective. For example, overexpression of DDR proteins such as ATM, DNA-PKcs, RAD51, or 53BP1 allows rapid repair of SBRT-induced DNA double-strand breaks, preventing lethal chromosomal damage and avoiding mitotic catastrophe [22,70]. Similarly, mutations in tumor suppressors like TP53 and SMAD4 disable apoptosis and senescence checkpoints, allowing cells to tolerate radiation-induced damage and repopulate the tumor [23,57,58]. Inflammatory signaling further reinforces resistance. Activation of NF-κB and STAT3 upregulates anti-apoptotic proteins and antioxidants, while cytokines such as IL-6 and TGF-β promote tissue repair and immune suppression [25,26]. Notably, these pathways are often interconnected. For instance, mutant p53 can directly promote inflammatory cytokine expression, and NF-κB activity can enhance both DDR gene expression and cell survival, generating a multifactorial resistance phenotype [3,17].

These insights have several translational implications:

1. Prediction of SBRT resistance: Radiogenomic profiling can identify tumors harboring mutations (e.g., in *TP53*, *KEAP1*) or gene expression patterns (e.g., high *PRKDC* or *RAD51*) predictive of poor response. Models such as Genomic-Adjusted Radiation Dose (GARD) use gene expression to estimate the biologic effect of radiation and suggest when dose escalation or combination therapy may be warranted [29].
2. Targeted radiosensitization strategies: Tumors with DDR upregulation may benefit from ATM, ATR, or DNA-PK inhibitors. Preclinical successes (e.g., ATR inhibitors in pancreatic cancer, ATM inhibitors in glioma) are now entering clinical trials [24,56]. In tumors with impaired apoptosis (e.g., *TP53* or *SMAD4* mutants), combining SBRT with BH3 mimetics or PARP inhibitors may induce synthetic lethality [64].
3. Personalized radiation dosing: Radiosensitive tumors (e.g., BRCA-mutant) may require lower doses, while resistant tumors may need dose intensification or combination with targeted agents [62,64].
4. Integration with immunotherapy: Tumors with high tumor mutation burden or intact STING signaling may benefit from SBRT-induced immune activation. Conversely, tumors with immune-evasive genotypes (e.g., *KEAP1*, *LKB1*) may require metabolic or inflammatory modulators (e.g., glutaminase inhibitors) in combination with SBRT and immunotherapy [13,17,33,36].

Tumor sensitivity to SBRT is not determined solely by dose or delivery precision, but rather by the intrinsic biology of the tumor. The field must advance toward prospective, biomarker-driven clinical trials. While much current evidence is preclinical or retrospective, new trials evaluate the impact of combining SBRT with targeted agents in genetically defined populations (e.g., ATM-mutated tumors with PARP inhibitors) [67,68,69]. Integrating genomic databases with SBRT outcomes will support the development of predictive algorithms and eventually real-time adaptive SBRT planning based on molecular changes.

## 6. Conclusions and Future Directions

Advances in radiogenomics are transforming our understanding of why some tumors respond dramatically to SBRT while others recur. Tumor response is shaped not only by physical dose and precision but by the tumor’s intrinsic biology—its DNA repair capacity, apoptotic potential, and interaction with the immune system. Radioresistant tumors frequently possess genetic alterations that enhance DNA repair, inhibit cell death, and create a protective inflammatory niche. These features can now be detected via genomic profiling, enabling prediction of SBRT response and guiding treatment intensification or modification. The future of SBRT lies in precision radiotherapy. Tumor sequencing may soon yield a radiosensitivity index or “radioresistance signature”, informing dose and drug selection. Simultaneously, developing radiosensitizers tailored to genetic vulnerabilities (e.g., *PARP* inhibitors for SMAD4- or *BRCA*-mutant tumors, ATR inhibitors for ATM-overexpressing cancers) will expand therapeutic options. Technological innovations will also drive personalization. Functional imaging may identify hypoxic or repair-active subregions for dose painting, and liquid biopsies could provide real-time insights into clonal evolution and treatment response. The concept of adaptive radiotherapy will evolve to incorporate not only anatomical but also molecular adaptation, modulating therapy based on dynamic biomarker changes during SBRT.

In conclusion, tumor genetics are critical to understanding, predicting, and overcoming resistance to SBRT. A “one-size-fits-all” approach to radiotherapy is no longer sufficient. Integrating radiogenic profiling, targeted radiosensitizers, and immunomodulation represents the next frontier. Multidisciplinary collaboration between radiation oncologists, molecular biologists, geneticists, and immunologists will be essential to realize the promise of precision SBRT fully. As ongoing trials continue to elucidate the best strategies, the trajectory is clear: tumor biology will guide the future of ablative radiation therapy.

## Figures and Tables

**Table 1:** Overview of stereotactic radiotherapy modalities.

Acronym	Full Term	Target Region	Typical Dose/Fraction	Number of Fractions	Common Clinical Indications
SABR	Stereotactic Ablative Radiotherapy	Extracranial (general term)	>5 Gy	1–5	Lung, liver, spine, prostate
SBRT	Stereotactic Body Radiotherapy	Extracranial (body)	>5 Gy	1–5	Lung, liver, adrenal, spine
SRS	Stereotactic Radiosurgery	Intracranial (brain/spine)	12–24 Gy (single fraction)	1 (occasionally 2–5)	Brain metastases, AVMs, vestibular schwannomas

**Table 2:** Key genetic alterations associated with SBRT radioresistance in pancreatic ductal adenocarcinoma (PDAC).

Gene	Frequency in PDAC	Mechanism of Radioresistance	Potential Inhibitor/Treatment
*SMAD4*	~55%	↑ ROS, ↑ autophagy, ↓ PARP1-mediated DNA repair	PARP inhibitors (e.g., olaparib) + RT
*TP53*	~75%	↓ apoptosis, ↑ inflammation, and immune suppression	MDM2 inhibitors (e.g., Nutlin-3a) + RT
*KRAS*	>90%	↑ NRF2/53BP1-mediated NHEJ, prevention of mitotic catastrophe	KRASG12C inhibitors (e.g., MRTX1133) + SBRT
*ATM*	2–18% (somatic)	Switch to alternative DNA repair pathways	ATR/PARP inhibitors
*BRCA1/2*	3–10%	↑ TMB, homologous recombination deficiency	Platinum-based chemotherapy, PARP inhibitors

↑ = increased; ↓ = decreased

**Table 3:** Tumor histology–specific SBRT outcomes: key genomic characteristics, and potential predictive biomarkers.

Tumor Histology	Key Genomic Characteristics	Potential Biomarkers
Soft -tissue sarcomas/melanoma metastases	Sarcomas: complex karyotype, increased expression of DNA repair and cell-cycle genes; Melanoma: BRAF/NRAS mutations, PTEN loss	Historically “radioresistant”—often require higher SBRT doses for equivalent control
Breast cancer metastases	Radiosensitive primary disease	Example of high efficacy with standard SBRT doses
Colorectal adenocarcinoma metastases	Intact MMR system, EGFR/AKT pathway activation	Tend toward chemoresistance and radioresistance
Hepatocellular carcinoma (HCC)	Common drivers: TP53, CTNNB1, TERT mutations; heterogeneous tumor genomics	*ADIPOR1*: ↑ post-SBRT in responders (100% sensitivity, 83% specificity) *EPB42*: expression changes correlate with outcome

↑ = increased

## List of Abbreviations

BED	Biologically Effective Dose	
CAF	Cancer-Associated Fibroblast	
CRC	Colorectal Cancer	
DDR	DNA Damage Response	
DNA-PK	DNA-Dependent Protein Kinase	
DSB	Double-Strand Break	
EGFR-TKI	Epidermal Growth Factor Receptor–Tyrosine Kinase Inhibitor	
GARD	Genomic-Adjusted Radiation Dose	
GWAS	Genome-Wide Association Studies	
HCC	Hepatocellular Carcinoma	
HR	Homologous Recombination	
IL-6	Interleukin-6	
LAPC	Locally Advanced Pancreatic Cancer	
LQ	Linear-Quadratic model	
MMR	Mismatch Repair	
NF-κB	Nuclear Factor kappa B	
NHEJ	Non-Homologous End-Joining	
NSCLC	Non-Small Cell Lung Cancer	
PARP	Poly(ADP-ribose) Polymerase	
PDAC	Pancreatic Ductal Adenocarcinoma	
PSMA	Prostate-Specific Membrane Antigen	
RCC	Renal Cell Carcinoma	
ROS	Reactive Oxygen Species	
SABR	Stereotactic Ablative Radiotherapy	
SBRT	Stereotactic Body Radiotherapy	
SRS	Stereotactic Radiosurgery	
TGF-β	Transforming Growth Factor-beta	
TNF-α	Tumor Necrosis Factor-alpha	
VEGF	Vascular Endothelial Growth Factor	
**Genes**		
ATM		Ataxia Telangiectasia Mutated
ATR		ATM and Rad3-Related
BRCA1		Breast Cancer 1
BRCA2		Breast Cancer 2
EGFR		Epidermal Growth Factor Receptor
KEAP1		Kelch-Like ECH-Associated Protein 1
KRAS		Kirsten Rat Sarcoma Viral Oncogene Homolog
LKB1		Liver Kinase B1
PTEN		Phosphatase and Tensin Homolog
RAD51		RAD51 Recombinase
SMAD4		SMAD Family Member 4
TP53		Tumor Protein 53
VHL		Von Hippel–Lindau
